# Does caste determine farmer access to quality information?

**DOI:** 10.1371/journal.pone.0210721

**Published:** 2019-01-25

**Authors:** Vijesh V. Krishna, Lagesh M. Aravalath, Surjit Vikraman

**Affiliations:** 1 Socioeconomics Program, International Maize and Wheat Improvement Center (CIMMYT), Texcoco, México CP, Mexico; 2 Department of Economics, ICFAI Business School (IBS) Hyderabad, Hyderabad, Telangana, India; 3 Centre for Corporate Social Responsibility, Public Private Partnership & Peoples Action, National Institute of Rural Development and Panchayati Raj, Hyderabad, Telangana, India; Shandong University of Science and Technology, CHINA

## Abstract

This paper explores the social inclusiveness of agricultural extension services in India. We estimate the probability and frequency of farmers’ access to extension services and resulting changes in crop income across different caste groups. The literature suggests that caste-based social segregation manifests in various spheres of life, and perpetuates economic inequality and oppression. An econometric analysis of nationally-representative data from rural India verifies this with respect to the agricultural sector. Farmers belonging to the socially-marginalized castes are found to have a lower chance of accessing the public extension services, primarily due to their inferior resource-endowment status. Contacting extension agents at least once increased the average annual crop income by about 12 thousand Indian rupees per household, which is equivalent to 36% of the annual crop income of those without access to extension services. There exists significant impact heterogeneity. Farmers from the socially-marginalized castes hardly benefited from accessing the extension services. Based on these observations, we have developed a number of policy recommendations that could improve the social inclusiveness of agricultural development strategies in rural India.

## Introduction

The dissemination of improved technologies forms an integral part of agrarian development in the Global South [1]. The speed of technology diffusion is determined by an array of socioeconomic and institutional factors, amongst which the human capital of farm households is particularly decisive. Agricultural extension is one of the important means to enhance human capital through the transfer of locally-relevant information from a global knowledge-base [2,1,3]. An examination of the Green Revolution literature indicates that the enhancement of crop productivity and rural livelihoods has been achieved not only through the increased use of material inputs, but also through the dissemination of information on crop production methods and farm management practices [4–7]. The relevance of information as an input to the agricultural production process has continued to increase over time, particularly in the context of the heightening risk and uncertainty from climate change and the degradation of the natural resource-base. While both formal and informal institutions disseminate information, often the informal ones are found to be inadequate to disseminate complex technologies, especially because the adoption process is sensitive to unobserved and intangible farmer characteristics and when the targeted communities are heterogeneous [8].

Outputs and outcomes of information disseminated by agricultural extension systems have significant public-good attributes, and hence, to find that most of the world's extension services are funded by government agencies is unsurprising [3]. Despite the non-excludability of services and deliberate governmental efforts, extension networks in many developing countries continue to have a strikingly low coverage of farmers [3,9,10]. For a faster and more inclusive agrarian growth and rural development, a clearer understanding of the process of farmer’s access to extension services is necessary. There have been studies on the differential access to and effectiveness of public extension systems in respect of farmers with varying farm size and education status [11], but not in respect of the social strata of the rural communities. In this paper, we examine the social inclusiveness of the agricultural extension system in India. Although India has one of the most heavily-invested, pluralistic extension systems in the public sphere [12,13], there is hardly any evidence in the literature on how the existing extension institutions and networks have evolved to address the concerns of the most vulnerable segments of the rural population in a sensitive manner.

Social inclusiveness is a crucial subject for developmental discourse [14]. With a rising concentration of wealth over the last two decades [15] and persisting economic disparity across different castes, religions, and gender [16–20], the concept of inclusion is of special relevance in Indian society. This study focuses on caste-based inequalities. The caste system, an exhaustive and hereditary institution, continues to permeate Indian society even today, clustering its population into thousands of endogamous groups [16,21,22]. We examine whether there is a differential access to the public extension services with respect to farmer caste, and explore possible impact heterogeneity of extension access across the caste groups.

The literature provides sufficient evidence for the persistence of caste disparities in different dimensions of rural livelihoods in India [19,23,24,18], although the complex mechanisms through which the caste system affects rural livelihoods have not been extensively studied. Farmers belonging to the castes and communities that are located on the lower rungs of the social hierarchy–henceforth the ‘socially-marginalized castes’–are found to have limited access to the factors of agricultural production, which could result in lower farm income [25–28]. Alongside the historical disadvantages with respect to their resource endowments, these farmers are often excluded from benefiting from rural development programs [29,30]. Caste is pivotal also in determining households’ access to public goods [31–33]. The Government of India has recognized the need to prioritize the socially-marginalized castes while framing agricultural strategies for technology dissemination [34]. Surprisingly, caste has not received sufficient academic focus in the context of dissemination of information, despite being an institution of immense significance for economic development, equality, and inter-group conflicts in rural India.

The existence of inter-caste disparities with respect to the access to and use of extension services can have far-reaching consequences. By resulting in unequal access to new farming technologies, and by restricting households’ adaptation strategies, these disparities reduce farm productivity as well as household income potential of the marginalized communities, making them more vulnerable to the vagaries of climate change. Against this backdrop, the present study examines the presence, prevalence, and economic effects of caste-based social segregation in accessing public agricultural extension services in India. We proceed by testing two hypotheses in this regard.

- The probability and frequency of farmers’ access to agricultural extension is lower for the socially-marginalized castes, and
- The incremental farm income from accessing public extension services is lower for the socially-marginalized castes.

Non-rejection of these hypotheses indicates that the socially-marginalized castes are in a disadvantaged position due to limited access to quality of information from the extension service in rural India.

In the next section, the background for the study is provided by briefly describing the extension approaches from the perspective of inclusive development. For the empirical analysis, we test the above-mentioned hypotheses by analysing data from a nationally-representative farm survey, conducted by the National Sample Survey Office, Ministry of Statistics and Programme Implementation of the Government of India. Descriptions of the survey and sample characteristics, alongside the econometric estimation procedure are shown in the following sections. Results from the econometric estimation are presented afterwards. The last section discusses the key findings and provides policy recommendations.

## Materials and methods

### Inclusiveness of agricultural extension programs in India

Several researchers have framed typologies of extension, based on the diverse philosophies, approaches, and strategies for the dissemination of agricultural information around the world. Providing assistance to households in making decisions regarding agricultural production by the transfer of information and technologies from researchers, and by educating households to refine their goals and possibilities, form some of the generic objectives of agricultural extension policies and programs [35]. In this section, we review the available literature to see how the different extension programme address social inclusion inadvertently or explicitly. Apart from caste and ethnicity, participation of women, marginal and landless households etc. comes under the purview of programme inclusion. Inclusive extension practices ideally remove institutional barriers and increase the access of diverse individuals and groups to development opportunities [36]. Since it is beyond the scope of the present study to compare and evaluate different national extension programmes for their inclusiveness, we briefly examine the available review papers and policy documents on inclusion determined by caste, and focus on information dissemination in Indian agriculture.

Social inclusion is a relatively recent development in policy research, and hence, unsurprisingly, not many studies have addressed this dimension while evaluating the performance of extension networks. One of the extensive reviews of literature on the impacts of extension, which covered 24 economic impact studies at the farm level and another 39 at the aggregate level, was published in 2001 [37]. None of the reviewed studies explicitly addressed the heterogeneity of the impacts. Neither has there been a significant change in recent years, with the possible exception of gender. Women’s empowerment and gender mainstreaming have been taken up extensively as topics of research [38–40]. Caste has not obtained similar academic attention, possibly because it is a social construct relevant only in the South Asian context. While some studies have indicated that farmers of the socially-marginalized castes have limited access to public extension services [25], the underlying reasons for this are left largely unexamined. As is the case with gender, caste mainstreaming–the process of ensuring that irrespective of their caste, people have equal access to production resources and developmental programs, while having control over decision-making in all stages of development processes–needs to be researched extensively to curb the caste-based inequalities in the South Asian countries.

Agricultural extension services in the Global South in general and India in particular are historically notorious for staff shortages and inefficient organizational structures for providing quality information at appropriate times [41,42]. The reach of extension services is even poorer among vulnerable and marginalized communities [25,43]. Farmers’ access to agricultural extension might not be uniform across households belonging to different castes due to social barriers. The usefulness of information obtained could also vary. The literature only provides some allusions as to how caste determines farmers’ access to extension services. The socio-political processes and networks that operate largely independently of the state may exclude farmers of the marginalized castes. Differential access to extension services could stem from differential access to resources such as irrigation water, land, capital and labour inputs [26,44,27]. Crops grown by small and marginal farmers–the groups that comprise mainly households from the socially-marginalized castes–are primarily grown for subsistence consumption, while the crops of larger farmers are of commercial interest, and this in turn leads to varying demand for information and differential returns [43]. Furthermore, the efficacy of extension could be affected by farmers’ education status. Illiteracy is prevalent among the socially-marginalized castes of rural India [22], limiting the scope of certain extension tools (e.g., pamphlets) to reach farmers of these groups.

An age-old observation on the functioning of the traditional extension system is worth reporting in this connection: “extension workers have tended to concentrate on the well-to-do farmers, because their efforts were more likely to produce an immediate and visible impact and because wealthier farmer could offer them personal benefits (meals, accommodation, produce)” [41]. Frequent social interaction between ‘contact farmers’–the direct recipients of a regular flow of information from extension personnel, that is to be passed on to others in the community–and the rest of the farming community is crucial for the diffusion of new technologies [45]. In heterogeneous farming conditions and hierarchical societies, information passed to the contact farmers does not necessarily reach every stratum of the society [8,46]. Caste-based social stratification increases the possibility of differential access to public extension systems. While there could arguably be some justification to selecting larger and wealthier farmers as the contacts on the grounds of productivity and efficiency, this bias could affect inclusiveness of the whole program and exacerbate economic inequality in the region.

In the more recent past, a number of novel extension institutions have been experimented with to increase the reach and efficacy of the provision of extension services in India. Decentralization of extension planning and monitoring, increased collaboration with non-governmental organizations (NGOs), and the formation of multidisciplinary teams of scientists, agro-clinics, and the Agricultural Technology Management Agency (a registered society for integrating the functions of key stakeholders involved in agricultural development) are some of the recent changes [47,42]. While social inclusion was not an explicit objective of these changes, they could inadvertently affect program participation of the marginalized. Nevertheless, these issues have escaped the radar of scholarly studies and hence we have only limited evidence. The present study could be one of the first attempts to assess the performance of the agricultural extension system from a caste perspective.

## Data

In 2013, a nationally-representative survey was conducted by the National Sample Survey Organization (NSSO) of the Indian government to assess the livelihood conditions of agricultural households. Validated household-level data, with actual village/ block identification masked, were available for public through a written request and payment of license free in 2015. This survey, called the “Situation Assessment Survey of Agricultural Households” (SAS 2013 henceforth), provides the database of our empirical analyses. Households with at least one member involved in farming activities, and generating a total value of produce of more than Indian rupees 3000 (≈USD 54) were included as the respondents. The sample contained households owning cultivable land and households farming on leased-in land [48]. The NSSO used a structured questionnaire to elicit information on socioeconomic, institutional, and organizational aspects of crop production as well as animal husbandry.

The SAS 2013 dataset contains information from two rounds of surveys that were conducted to collect relevant information for the two major agricultural seasons separately. The first visit was made during January to July 2013, and covered 4,529 rural villages from 625 districts. On an average, 8 households were selected in each sample village, making a total sample size of 35,200 households. Of these, 34,907 were revisited and surveyed in the second round. Information on expenses and receipts for crops and livestock were collected for the period July to December 2012 (*kharif* season) in the first visit, and for the period January to June 2013 (*rabi* season) in the second visit. For the present study, we excluded households having (i) no crops under cultivation in both *kharif* and *rabi* seasons, (ii) incomplete information, and (iii) extreme values in the crop income distribution (top 1% and bottom 1%). The resulting dataset contained information from 31,181 farm households. The dataset with the variables used in the analysis is available as S1 File.

In the survey design, NSSO employed a concept of interpenetrating sub-samples. For the surveys in the rural sector, from where the SAS 2013 dataset developed, the Organization followed probability proportional to size sampling strategy, in which the number of households sampled from a region depended on the total population of that region. The samples within the sub-sample were drawn independently. These sub-sample estimates were then combined together across the regions. After the unit level dataset was finalised, the multiplier values or sampling weights were calculated as per the sampling design by the NSSO in a manner such that simple aggregation could generate nationally representative estimates. These weights were posted in the unit level dataset by NSSO, which we used in the analysis to make our estimates representative at the national level. Further details on survey design and sampling is available online at http://www.icssrdataservice.in/datarepository/index.php/catalog/105 (Accessed on 31 October 2018).

In SAS 2013 questionnaire, respondents were asked to state their caste and religion. The survey data did not include the caste name *per se*, but provided four collectively exhaustive and mutually exclusive caste categories–non-marginalized castes, scheduled castes, scheduled tribes, other socially-marginalized communities (OSMC). Scheduled castes and tribes were the formerly disadvantaged communities, for whom the Constitution of India allows for special provisions [22,29]. According to the recent census data, about 25% of the Indian population belongs to these two categories [49]. The term ‘other socially-marginalized communities’ corresponds to ‘other backward classes’ (OBC), a term officially used by the Indian Government, which we found potentially stigmatizing. Details on these caste groups, including their relative social position, are provided by a number of studies in the past [50,51], making a reiteration obsolete. In this paper, OSMC households belonging to the Islam religion were grouped separately, due to the relative deprivation of this community reported in many spheres of life, such as education, employment, and participation in government programs [52]. The population share of OSMCs has not been revealed in the recent census. In the SAS 2013 dataset, about 41% of households belonged to OSMC non-Muslim and 4% to OSMC Muslim. The caste composition shows significant inter-state variation.

## Analytical framework

The objective of our study is not only to verify whether caste determines farmer access to quality extension services, but also to find out why. Does differential access to quality extension services arise from social exclusion (a direct effect of caste system), or from the differences in resource endowment status of marginalized castes (an indirect effect)? To answer this question, the regression analysis was carried out in two steps–basic and extended models were estimated to explain farmer access to extension services and associated income effects. Basic models included mainly the caste dummies (binary variables representing marginalized castes) as the explanatory variables, while the extended models were with an array of socio-economic factors in addition, representing the differential resource endowments and production constraints of farm household. The coefficients of caste dummies in the basic models would capture both the direct and the indirect effects of cast system. In the extended model, as we included variables standing proxy for households’ resource endowments and production constraints as the explanatory variables, direct effects are separated out from the indirect ones. If the coefficients of caste dummies are significant both in the basic and extended models, there is a high possibility for caste-based exclusion in the extension networks. If they are significant only in the basic models, historical disadvantages with respect to their resource endowments are the main reason for lower access and benefits from extension services.

### Identifying the determinants of access to public extension

To test Hypothesis [I], that ‘the probability and frequency of farmers’ access to agricultural extension is lower for the socially-marginalized’, regression models are estimated with extension contact as the dependent variable and farmer caste dummies and socioeconomic attributes as the explanatory variables. Farmers’ access to formal extension services is captured with two variables–a binary variable for households that had made contact with the extension agents at least once, and the total number of times a household had contacted the extension agents (frequency of contact) during the previous two cropping seasons. In this paper, the term extension contact denotes either a visit by extension personnel (from state extension, *Krishi Vigyan Kendras* or Agricultural Science Centres, or state agricultural universities), or a visit by farmers to any of these institutions or the meetings and field demonstrations organized by these institutions. Modelling farmer access is a necessary predecessor for estimating the income effects of extension. Because more frequent contacts might result in better crop management and thus increased crop income, the factors affecting contact probability and frequency needs to be studied separately.

We use probit regression to model contact dummy, and count-data regression to model frequency of contact. As probit models are widely used in the literature to study household decision-making, we are not elaborating the specification. However, modelling the frequency of contact is not straightforward. In SAS 2013 dataset, about 89% of households did not access formal extension during the study period, leading to an “excess” of zero values in the contact frequency variable. Classic count-data models would generate biased estimates if the dependent variable has an excess of zero counts [53]. Zero-inflated count estimators provide a parsimonious yet powerful way to model such variables. In this study, we have attempted zero-inflated Poisson (ZIP) and zero-inflated negative binomial (ZINB) specifications. Both assume that data are a mixture of two separate data generation processes–one generates only zero values and the other is either a Poisson or a negative binomial data-generating process. The selection of ZIP/ ZINB over conventional Poisson and negative binomial models was also supported by positive and significant Vuong test statistics [54]. A comparison of ZIP and ZINB estimates was carried out using Akaike's information criterion and Bayesian information criterion, which showed a clear superiority of ZINB to explain the variation in the dependent variable. Through a splitting process that models the outcomes as zero or non-zero, the ZINB framework combines a negative binomial regression model with a binary model [55]. Unlike ZIP, over-dispersion is also allowed in ZINB, that is, when the conditional variance exceeds the conditional mean of the distribution. The ZINB models have been used to explain determinants of count outcomes in a number of empirical studies in different contexts [56–58].

### Modelling the differential effects of extension

To test Hypothesis [II], that ‘the incremental farm income from accessing public extension services is lower for the socially-marginalized castes’, we quantified the effect of extension contact after categorizing the sample households into two groups–those who accessed, and those who did not access the extension services during the study period. As the first step in this direction, ordinary least squares (OLS) regression models were estimated with crop income as the dependent variable and dummy variables representing extension and access and caste groups as the explanatory variables. Crop income was calculated by aggregating income from all crops grown by the household during the study period, and shown in thousand Indian rupees per household per year. For the parameter estimates, clustered standard errors at the district level were used. Many of the extension programs are designed and implemented at the district level (e.g., agricultural technology management agency or ATMA [59]), and extension agencies might be more efficient in some districts than others, potentially leading to intra-district correlation of the model errors. Clustering the sample errors will allow for intra-district correlation, relaxing the usual requirement of OLS models that the observations be independent [60].

#### Establishing causality

The causal effect of extension access on crop income is not easy to quantify [11,10]. While the OLS models are simple to understand and interpret, and popular in the academic literature, they are inadequate to establish causality. This is because the treatment variable (extension contact) could be subject to endogeneity bias [61,62], and if so, correction is required to yield unbiased estimates. While modelling the effects of farmer access to public extension services, endogeneity bias may arise from three major sources.

- Extension agencies may decide to concentrate their efforts on highly productive regions to obtain quickly visible results (endogenous program placement).
- Extension personnel responsible for a given village might be contacting better-endowed and more efficient farmers frequently in order to meet the program goals.
- Because many of the extension contacts are initiated by farmers, several unobserved individual and household attributes could be determining both household’s demand for information and use of this information to augment crop income [41].

To eliminate the bias from source (i), we incorporate regional fixed effects in the regression analysis. The statements (ii) and (iii) indicates two potential sources of household-level endogeneity, which could lead to biased estimates. To address this, the effects of extension access are modelled in an endogenous treatment-effects (ETE) framework, with bias-corrected matching estimator. This allows us to estimate the average treatment effects–the change in an outcome by getting one treatment instead of another–from observational data. The goal of the ETE estimators is to utilize covariates to make treatment and outcome independent once we condition on those covariates [63,64]. The estimation involves two stages. The first stage is a selection equation that models the determinants of access to public extension. In the second stage, regime equations are specified explaining the outcome of interest (crop income) based on the estimated selection function. The estimation procedure in ETE uses a linear model for the outcome variable, and a normal distribution to model nonconformity to the assumption of conditional independence imposed by the estimators. In this approach, the endogenous binary-variable model allows for a specific correlation structure between the unobserved variables that affect the treatment (i.e., access to extension) and the unobserved variables that affect the potential outcome (i.e., crop income) [65]. A number of studies have used ETE models to estimate the impact of technology adoption in agriculture [66,67].

For the ETE model to be correctly specified, the selection equation should contain at least one variable that is significant in the selection model but not directly correlated with the outcome variable. We use the extent of non-availability of extension services in the district as this instrument. The reasons for not accessing extension services were elicited in the SAS 2013 questionnaire, and included lack of awareness about the available services, non-availability of the services, and lack of interest (“services not required”). While lack of awareness and interest are farmer-specific attributes, non-availability of extension services is determined largely in the supply-side institutions and hence is exogenous to the farm household. We estimated non-availability as the share of households in a district that did not access public extension because of the non-availability factor. Similar variables at the supply-side were employed to correct endogeneity bias in previous studies on the impacts of information access and technology adoption [68,62]. Although this variable is neither farm-specific nor endogenous, one could still criticise its selection as an instrument, as districts with low agricultural potential might have received poorer developmental focus. Due to this reason, non-availability of extension might be correlated with the overall agricultural potential of the region, affecting the performance of individual farmers indirectly, and thus weakening the assumption of exclusion restriction associated with the instrument selection. However, if this argument holds true, the non-availability variable will be strongly (and negatively) correlated with the farm income of households who did not have any extension access. Following the procedure employed by Di Falco and colleagues [69], we ensured that the non-availability variable is not correlated with the crop income of households who did not access extension services, and hence qualifies to be included in the ETE framework.

Besides estimating the impact at the aggregate level, ETE models were re-estimated for the five caste groups. However, this resulted in a significant reduction in the sample size, which could lead to lack of statistical power to estimate the treatment effects. To check the robustness of the estimates and to verify the presence of the heterogeneous impacts of extension across caste groups, we also employed non-parametric propensity score matching (PSM). Here, we first controlled for all the observed factors that could affect access to extension services and based on the estimated propensity score, matched households that had accessed extension services with households that had not. This approach is relatively simple and widely employed to study the effects of technology adoption in agriculture [70–73].

To estimate the effects of extension access with PSM, we first specified the conditional probability using a probit model. In the second step, farm households that had accessed extension services were matched with those that had not, based on similarity in their propensity scores. In order to match technology adopters with non-adopters based on their distribution of observed attributes, a number of algorithms have been proposed in the literature [74]. Following the popular practice, we employed ‘kernel-based matching’ and ‘nearest-neighbour matching’. In kernel-based matching, weighted averages of outcomes of all households that did not access extension services are used to construct the counterfactual. These weighted averages are inversely associated with the distance between propensity scores [75]. While nearest-neighbour matching involves choosing farmers accessing extension and not accessing extension that are closest in terms of propensity score, as a matching pair. This is usually applied with replacement so that the control sample can be the best-matched pair for more than one treated sample [76].

## Results

The mean-variance analysis indicates significant differences in the key socioeconomic variables of sample households from the four socially-marginalized caste groups (scheduled castes, scheduled tribes, OSMC Muslims, and OSMC non-Muslims), when compared with the non-marginalized castes (Table 1). As anticipated, the average crop income realized by non-marginalized castes was significantly high. The magnitude of the difference is the highest (134%) with the scheduled castes. The difference with other marginalized-caste groups are also high and statistically significant at the 0.01 level. These crop income calculations are made by using only the paid-out costs, as imputed costs were not elicited in SAS 2013. A large share of socially-marginalized caste farmers, due to poverty, could be less dependent on the factor markets and more on family labour and owned animals for crop production. Inclusion of imputed costs in the estimation might hence widen the inter-caste differences in income.

**Table 1:** Descriptive statistics by caste groups.

Farm household characteristics [unit of measurement]	Pooled	Castes groups				
		Scheduled castes	Scheduled tribes	OSMC Muslim	OSMC non-Muslim	Non-marginalized
Extension contact [dummy]	0.11	0.07**	0.10**	0.08**	0.11**	0.13
Frequency of extension contact [number]	0.76	0.48**	0.63**	0.51**	0.74**	0.94
	(0.03)	(0.03)	(0.03)	(0.05)	(0.02)	(0.03)
Landholding [ha per adult equivalent]	0.37	0.23**	0.37**	0.23**	0.39**	0.43
	(0.01)	(0.01)	(0.01)	(0.01)	(0.01)	(0.01)
Household size [adult equivalent]	2.79	2.68**	2.76	3.05**	2.80**	2.75
	(0.02)	(0.02)	(0.01)	(0.03)	(0.01)	(0.01)
Education of household head [scale; 1–13]	4.58	3.99**	3.65**	3.78**	4.50**	5.72
	(0.06)	(0.05)	(0.04)	(0.09)	(0.03)	(0.04)
Main income source of the household is off-farm [dummy]	0.04	0.04**	0.02**	0.08**	0.04**	0.05
Household is a ‘Below Poverty Line’ (BPL) card holder [dummy]	0.47	0.63**	0.72**	0.38**	0.44**	0.31
Crop income [‘000 Indian rupees/household/year]						
a. Overall	36.02	20.13**	29.75**	33.55**	37.51**	47.20
	(0.66)	(0.98)	(1.04)	(5.66)	(1.04)	(1.55)
b. Households having extension contact	56.38^#^	33.24^#^^,^**	44.46^#^^,^**	38.63**	54.88^#^^,^**	73.13^#^
	(1.18)	(2.48)	(2.01)	(5.08)	(1.87)	(2.50)
c. Households without extension contact	33.59	19.11**	28.11**	33.13**	35.38**	43.33
	(0.35)	(0.61)	(0.62)	(1.73)	(0.58)	(0.86)
Number of observations	31,181	3,675	6,203	1,225	11,314	8,764

Notes: Mean values are shown with std. errors in parentheses. Sampling weights given in the SAS 2013 database are employed in the estimation.

**Difference with the mean value of ‘non-marginalized’ category is statistically significant at 0.01 level.

^#^Difference with the mean value of ‘households without extension contact’ category is statistically significant at 0.01 level.

1US$ = 58.6 Indian rupees in 2013 (source: [77]). OSMC stands for ‘other socially-marginalized communities’.

One of the possible reasons why marginalized castes obtain lower crop income is their lower access to public extension networks. Overall, the reach of public extension in Indian agriculture is limited in India, with only 11% of farm households accessed the services in 2013. When compared with the access rate from the Situation Assessment Survey conducted in 2003 [25], no significant improvement in the reach of extension system was observed. During 2013, only 7% of the scheduled-caste farmers had access to public extension services, compared to 13% of the non-marginalized castes (Table 1). Farmers belonging to the marginalized castes have been disadvantaged with respect to other production resources also. An analysis of SAS 2013 data indicates that farm households belonging to the scheduled castes and OSMC Muslim category possessed smaller landholdings (0.23 ha per adult equivalent) compared to the non-marginalized castes (0.43 ha per adult equivalent). In the literature, there is ample evidence that land ownership is perpetually skewed against the socially-marginalized, critically limiting their potential for income generation [17,27,48]. Significant differences also exist with respect to human capital. Low educational attainment of the socially-marginalized castes is evident in the sample, as in the literature [17,22,52].

As a cumulative effect of historic disparities with respect to ownership of production resources and social and physical exclusion, farm households of the socially-marginalized castes are economically poor [22]. This is reflected in their increased participation in the food-subsidy schemes meant for households living ‘Below the Poverty Line’ (BPL). About 63% of scheduled-caste households and 72% of scheduled-tribe households in the sample were recipients of BPL food subsidies, against only 31% in the non-marginalized (Table 1).

Access to extension services is associated with higher crop income across all caste groups. Overall, the farmers with extension access realized 68% higher crop income. This difference in percentage terms is the highest for scheduled castes (74%) and scheduled tribes (58%). In absolute terms, the difference is the highest for the non-marginalized castes. If the observed income differences translate into causality, extension can be counted as a crucial instrument for alleviation of rural poverty in India. However, a number of socioeconomic factors could be determining the farmer access and income generation potential of public extension, which are examined in detail in the next sub-sections.

### Differential access to public extension services

Determinants of farmer contact with public extension services and frequency of contact are measured as dichotomous and count variables respectively. The extension contact dummy was the dependent variable in probit models, and frequency of contact in ZINB models. Both types of models are estimated in two sets. In the first set, we modelled contact dummy and frequency only with caste and regional variables. Here, if the coefficients of caste dummies are negative and statistically significant, it could be due to social exclusion and/or differential production constraints. To control for the effect of production constraints, a second set of models were estimated including more household-specific attributes. Statistically significant and negative caste coefficients in Model 2 denote that farmers of marginalized castes could be facing certain social exclusion for accessing information from the public extension networks. The marginal effects of caste dummies and other variables are reported in Table 2.

**Table 2:** Determinants of farmers’ access to public extension services in India.

	Model 1		Model 2	
	(a) Probit	(b) ZINB	(a) Probit	(b) ZINB
*Caste categories [dummy; reference*: *non-marginalized castes and communities]*				
Scheduled castes	-0.057**	-0.457**	-0.023	-0.207
	(0.013)	(0.115)	(0.012)	(0.108)
Scheduled tribes	-0.063**	-0.572**	-0.032	-0.350**
	(0.017)	(0.135)	(0.017)	(0.133)
OSMCs, Muslim	-0.082**	-0.675**	-0.058*	-0.485*
	(0.024)	(0.206)	(0.024)	(0.192)
OSMCs, non-Muslim	-0.034**	-0.285**	-0.018	-0.169
	(0.011)	(0.090)	(0.011)	(0.087)
*Farm-household characteristics*				
Homestead farming [dummy]			-0.022	-0.214
			(0.025)	(0.170)
Size of land owned [ha per adult equivalent]			0.032**	0.192**
			(0.008)	(0.049)
Household size [adult equivalents]			0.010**	0.081**
			(0.004)	(0.025)
Household head’s age [years]			0.001**	0.007**
			(2.9E-04)	(0.002)
Female household head [dummy]			-0.015	0.006
			(0.013)	(0.174)
Household head’s education [scale; 1–13]			0.008**	0.067**
			(0.001)	(0.011)
Possesses owned dwelling [dummy]			-0.031	-0.269
			(0.026)	(0.187)
Type of dwelling [1 = bad/ kaccha, 2 = medium/ semi-pucca 3 = good/ pucca]			0.008	0.021
			(0.007)	(0.065)
*Off-farm income sources [dummy variables]*				
Livestock production			-0.042*	-0.329*
			(0.019)	(0.144)
Non-farm employment			-0.033	-0.357**
			(0.019)	(0.137)
Wage employment			-0.030**	-0.329**
			(0.012)	(0.089)
Pension and remittance			-0.044	-0.445*
			(0.027)	(0.212)
*Region [dummy; reference*: *North India]*				
East India	0.052**	0.455**	0.066**	0.540**
	(0.018)	(0.161)	(0.018)	(0.152)
South India	0.268**	1.935**	0.267**	1.965**
	(0.028)	(0.201)	(0.028)	(0.208)
Semi-Arid Tropics	0.126**	0.790**	0.125**	0.775**
	(0.018)	(0.144)	(0.017)	(0.137)
Rest of India	0.115**	0.770**	0.118**	0.798**
	(0.029)	(0.180)	(0.028)	(0.181)
*Caste dominance at the district level [dummy]*				
Non-marginalized castes form a majority [i.e. farmer is from a district where ≥67% belong to the non-marginalized castes]	0.042*	0.423**	0.044*	0.442**
	(0.021)	(0.150)	(0.021)	(0.151)
Socially-marginalized castes form a majority [i.e., farmer is from a district where ≥67% belong to the marginalized castes]	0.043*	0.476**	0.041*	0.450**
	(0.018)	(0.133)	(0.018)	(0.129)
Number of observations	31,181	31,181	31,153	31,153
Wald chi^2^	205.988	65.287	450.870	115.653

Notes: Marginal effects are reported with standard errors clustered at the district-level in parentheses. Sampling weights given in the SAS 2013 database are employed in the estimation. The dependent variable in the probit models is extension contact (dummy) and in the zero-inflated negative binomial (ZINB) models is the frequency of contact (number per year).

*, **: Statistically significant at 0.05 and 0.01 levels, respectively.

OSMC stands for ‘other socially-marginalized communities’.

In the first specification in both probit and ZINB models, the marginal effects of all caste dummies were negative and statistically significant at 0.01 level, indicating that both probability and frequency of access are lower for farmers belonging to marginalized castes. Compared to farmers of non-marginalized castes, the probability of accessing extension services was lower by 3.4% points for OSMC non-Muslim, 5.7% points for scheduled castes, 6.3% points for schedule tribes, and 8.2% points for OSMC Muslim category. Considering that farmer access to extension in India is generally low, these inter-caste differences are highly detrimental for inclusive agrarian development. For instance, the likelihood that a farmer from one of the marginalized castes would contact an extension agent was 26–63% lower compared to one from non-marginalized caste. Similar patterns are observed in the frequency models also. Compared to the non-marginalized, the frequency of contact was lower by 0.29 events (-30% compared to the non-marginalized) for OSMC non-Muslims, 0.46 events (-49%) for scheduled castes, 0.57 events (-60%) for scheduled tribes, and by 0.68 events (-71%) for OSMC Muslims.

Inclusion of the socioeconomic attributes of farm households in the model reduces the magnitude of the effect of caste dummies, and the differences become statistically insignificant for most caste groups. Compared to the non-marginalized, probability of access was by 5.8% points for OSMC Muslims (model set 2a). The count-data models on frequency of farmer contact with extension agents showed negative and significant effects for scheduled tribes and OSMC Muslims. These differences have a significant policy relevance since we have already controlled for size of landholding, education, sources of off-farm income, and the general in of sample households. This means that while the inferior resource endowments of the socially-marginalized castes form a major constraint in accessing extension services, we cannot reject the hypothesis that their caste *per se* has a role in defining access to extension services for some of the caste groups. Nor can we overlook the fact that attributes such as farm size and education, where the socially-marginalized castes are historically disadvantaged, were the major factors determining farmer access to extension services.

Farmer access to extension services also depends on the demographic composition of the region where they reside. In districts where the marginalized and non-marginalized had a more or less comparable population share (heterogeneous population with respect to caste), farmers’ access was the lowest. Surprisingly, there was no significant difference between the districts where marginalized households formed a majority and the districts where the non-marginalized formed a majority.

### Heterogeneous impacts of extension contact

In this section, we examine the effect of farmers’ access to extension services on crop income, using both parametric and non-parametric methods. As the first step, we employ OLS regression models on crop income with the key “treatment” variable–i.e., extension contact–included either in binary or in count form. Although OLS can only provide information on the association between extension access and income, and not causality, we will get simple and easy to understand relationship patterns. In these models, extension variables are interacted with caste dummies. Statistically-significant caste-extension interaction terms denote the heterogeneity of the impact of public extension networks. The model estimates are presented in Table 3 and the full model in S1 Table.

**Table 3:** Caste-differentiated effects of extension contact on crop income: OLS regression estimates.

	Model 1		Model 2	
	(a) With extension dummy	(b) With extension frequency	(a) With extension dummy	(b) With extension frequency
*Caste categories [dummy variables; reference*: *non-marginalized castes]*				
Scheduled castes	-25.195**	-25.462**	-7.836**	-7.946**
	(2.382)	(2.364)	(1.892)	(1.887)
Scheduled tribes	-16.007**	-16.164**	-5.947*	-5.740*
	(3.080)	(3.037)	(2.463)	(2.421)
OSMC Muslim	-7.768	-8.129	-1.311	-1.339
	(6.710)	(6.651)	(4.814)	(4.760)
OSMC non-Muslim	-10.656**	-10.593**	-3.680*	-3.642*
	(2.392)	(2.326)	(1.826)	(1.788)
*Extension [dummy or frequency] and caste interaction terms*				
Extension	28.228**	3.648**	12.511*	1.905**
	(5.867)	(0.759)	(5.228)	(0.616)
Scheduled castes x Extension	-15.276*	-1.554	-8.276	-0.840
	(6.457)	(0.947)	(6.117)	(0.839)
Scheduled tribes x Extension	-11.839	-1.225	-5.090	-0.716
	(7.464)	(0.985)	(6.268)	(0.767)
OSMC Muslim x Extension	-25.120*	-2.466	-15.559	-1.721
	(12.133)	(1.683)	(9.186)	(1.275)
OSMC non-Muslim x Extension	-8.474	-1.150	-4.242	-0.528
	(6.597)	(1.010)	(5.861)	(0.794)
*Caste dominance at the district level [dummy]*				
Non-marginalized castes form a majority [i.e. farmer is from a district where ≥67% belong to the non-marginalized castes]	11.637	11.237	12.022*	11.717*
	(6.258)	(6.227)	(4.922)	(4.904)
Socially-marginalized castes form a majority [i.e., farmer is from a district where ≥67% belong to the marginalized castes]	5.145	4.695	4.080	3.760
	(2.667)	(2.620)	(2.120)	(2.105)
Number of observations	31,181	31,181	31,153	31,153

Notes: Coefficients are shown with std. errors clustered at the district level in parentheses. Sampling weights given in the SAS 2013 database are employed in the estimation. The dependent variable across the models is crop income (measured in thousand Indian rupees; 1US$ = 58.6 rupees in 2013 (source: [77])). Model 1 includes caste and regional dummy variables only, and Model 2 includes farm household characteristics and types of crops cultivated additionally. See S1 Table for the full models.

*, **: Statistically significant at 0.05 and 0.01 levels, respectively.

OSMC stands for ‘other socially-marginalized communities’.

The effect of extension on crop income is estimated first by including only caste and regional dummies, without other household-specific variables (Model 1). The coefficient of the extension contact dummy was positive and statistically significant at 0.01 level. Among farmers of non-marginalized castes, those who accessed extension services are found to realize a higher crop income (about 28 thousand Indian rupees or US$ 482 per household annually) compared to those without extension access. In the model with extension frequency, the effect was 3 thousand Indian rupees (US$ 62) per additional event of contact. All the coefficients of caste dummies and their interaction with extension are negative in Models 1a and 1b. In comparison to non-marginalized castes, scheduled caste and scheduled tribe households not only generated significantly lower crop income on average, but also could benefit only marginally from accessing the extension services. The OSMC Muslim households did not realize any incremental income through extension contact. For the OSMC non-Muslim category on the other hand, the benefits of extension contact was comparable to that of the non-marginalized caste. In Model 1b, where extension contact was measured as the frequency, the caste-interaction terms were statistically insignificant.

When the variables representing farm households’ endowments were included in the model estimation, the marginal returns to extension contact diminished drastically across all caste groups (Models 2a and b, Table 3). Also most of the caste-extension interactions became statistically insignificant. The marginal effect of access to extension, as estimated from Model 2a, was 13 thousand rupees per annum. When district-level dummy variables were included in the estimation (provided in S2 Table), the coefficient of the extension access variable reduced even further in magnitude. The reduction in magnitude of the extension access dummy alongside negative interaction terms denotes that most of socially-marginalized households were not at all benefiting from the public extension networks. The major reason is not social exclusion but the lower resource-endowment status, such as having smaller farms and lacking formal education.

The reduction in the effect of extension contact after including district-level dummy variables denotes the importance of supply-side factors affecting the availability of information. The district-level extension infrastructure and programs in the public sector could vary depending on a number of factors, including the caste composition (demographic share of different castes) of the district. We test possible differences in the quality of extension services by employing additional regression models, segregating the districts based on demographic composition of caste and poverty. The key estimates from OLS models are shown in S3 Table. The results show two highly striking patterns.

1. In districts with a small share of marginalized communities and poor households (<33% in the sample), the non-marginalized castes benefited significantly (19–29 thousand rupees per year) from public extension. The marginalized castes did not benefit at all.
2. In districts with a large share of marginalized castes and poor households (>66% in the sample), there was no benefit from extension for any caste groups.

These estimates provides direct evidence for discrimination occurring through differential quality of public extension services based on demographic composition of districts.

Abovementioned OLS regression estimates provide direct, easy-to-observe measures of the utility of extension services, although they could be criticized for endogeneity or omitted variable bias. The presence and magnitude of this bias can be addressed through an endogenous treatment effects (ETE) model that includes an instrumental variable, the non-availability of extension services in the district. Non-availability is measured as the share of farmers who did not access extension services due to non-availability of these services in their locality. While this variable is strongly correlated with farmer’s decision to contact extension personnel, it does not affect the outcome variable in the group of households without access to extension (S1 Fig. and S4 Table). For the robustness check, the effects of extension on crop income were additionally captured using non-parametric PSM. The rationale of employing multiple approaches to capture the impact of extension is described in the methodology section.

Table 4 includes both ETE and PSM estimates, which are comparable in magnitude. The effect of extension contact is statistically significant for all caste groups except the OSMC Muslim category. Overall, by accessing extension services farmers generated an incremental income of about 12–13 thousand rupees annually. Similar to the OLS estimates, non-marginalized farmers benefited the most (17 thousand rupees in ETE and 21 thousand rupees in PSM). Among scheduled caste, scheduled tribe, and OSMC non-Muslim households, the incremental income from accessing extension services was small (7–13 thousand rupees). In tune with the regression estimates, OSMC Muslim households did not benefit at all.

**Table 4:** Treatment effect of extension contact.

	ETE (all India)	PSM (all India)	Effects from PSM estimates in districts where			
			Non-marginalized communities form a majority [>66%]	Marginalized communities form a majority [>66%]	Non-BPL households form a majority [>66%]	BPL households form a majority [>66%]
Overall	13.287**	12.352**	50.354**	7.099**	15.514**	7.946**
	(1.762)	(1.527)	(5.9458)	(1.707)	(2.465)	(2.819)
Scheduled castes	7.240*	13.983**	36.542	10.513**	15.914**	9.506
	(3.332)	(3.716)	(23.163)	(3.938)	(5.834)	(6.358)
Scheduled tribes	10.228**	10.927**	113.276	12.122**	9.813	4.749
	(3.105)	(2.828)	(59.555)	(2.952)	(6.348)	(4.369)
OSMC Muslim	7.862	-2.236	-84.558	-9.882	-1.896	-0.582
	(7.866)	(8.861)	(48.678)	(10.778)	(10.384)	(25.080)
OSMC non-Muslim	9.955**	7.129**	59.472**	8.339**	5.813	7.749
	(2.659)	(2.411)	(18.068)	(2.739)	(3.957)	(4.332)
Non-marginalized communities	16.584**	21.173**	51.042**	-2.451	28.100**	9.527
	(3.049)	(3.164)	(6.6749)	(4.571)	(4.260)	(7.740)

Notes: Average treatment effects of extension access (dummy variable) on crop income (thousand Indian rupees; 1US$ = 58.6 rupees in 2013 (source: [77]) are shown in the table with std. errors in parentheses. Sampling weights given in the SAS 2013 database are employed in the estimation.

*, **: Statistically significant at 0.05 and 0.01 levels respectively.

OSMC stands for ‘other socially-marginalized communities’ and BPL for ‘below poverty line’.

We repeated the PSM analysis after segregating the study districts based on the demographic composition of caste and poverty. The key estimates are shown in Table 4. The results show patterns similar to that in the OLS estimates. In districts with a small share of marginalized castes and poor, the non-marginalized communities benefited significantly from the public extension networks. While the observed effects are positive for the marginalized communities (except OSMC Muslims), many are statistically insignificant. In districts with a large share of marginalized communities and poor, the incremental income from contacting public extension networks was small. These findings indicate that we cannot reject the hypothesis (ii) on the heterogeneous income impacts of extension. The constraints to the access to and use of extension services exist in both demand and supply sides. Caste continues to define the economic opportunities for a vast proportion of the farming population in rural India, and our results are consistent to that of previous studies [18,17].

## Discussion

With the support of empirical evidence, the present study reiterates the commonly-held perception that caste as a social institution influences the economic outcomes in rural India, and shows that differential access to quality information is one of the main pathways. Our analysis of nationally-representative data shows that farmers who received at least one extension visit increased crop income by about 12 thousand rupees (US$ 205) per year, which is about 36% of the crop income of those who did not access any extension services. However, this benefit was not universal; farmers of the socially-marginalized castes benefited either by a smaller amount or did not benefit at all from extension access. While discrimination based on group attributes such as ethnicity and gender has long attracted the attention of socio-economists [78–80], there exists only limited empirical evidence on caste-based social segregation that shape agrarian relations and economic outcomes. We conclude that farmers of the socially-marginalized castes are economically-disadvantaged due to three factors: (1) inferior resource endowment status, (2) exclusion from public extension networks, and (3) and regional differences in the quality of extension services and infrastructure. Availability of timely and quality information on crop production technologies could help these farmers escape the poverty trap. Ironically, one of the major reasons for lower access to formal extension services is the monetary constraint faced by the socially-marginalized castes and communities, forming a vicious, self-enforcing mechanism for the persistence of poverty.

A number of studies and reports have indicated that agricultural extension in India is underinvested. The average spending on agricultural education, research, and extension (ER&E) for 2014–15 was only 0.70% of the annual agricultural gross domestic product of India, much below the optimal 2% set by the World Bank [59]. Nevertheless, the assumption that just by increasing the public investment would inevitably ensure socially-inclusive agricultural growth requires a major revision.

Agricultural extension in India has been undergoing a series of institutional changes to decentralize the existing governance framework, in order to allocate available resources more effectively to address constantly-evolving social demands. Decentralization of extension activities alone may not necessarily ensure inclusion, especially since the new system could also suffer from lack of accountability and political commitment. Ignoring caste identities and inter-caste heterogeneities could pose serious challenges for inclusive growth. Our study has shown that economic inequalities among farm households of various social groups in rural India emerge largely from caste inequalities in ownership and access to means of production and technology. Research solely based on the economic stratification of society, that neglects social dimensions and especially that of caste, might lead to ineffective policy recommendations. Explicit and focused efforts to ensure adequate participation of the socially-marginalized in developmental programmes are fundamental for inclusive growth. Equally important is to recognize the specific requirements for information and production constraints that are unique to each of these castes and communities. Against this backdrop, following policy recommendations are framed to facilitate an inclusive extension strategy.

### Policy recommendations

More focused investment in public extension: One the root causes of low coverage of agricultural extension in India is the sub-optimal public spending, which also shows significant regional variation. Particularly in those states, where significant share of the population belongs to the marginalized castes, the spending on agricultural ER&E is much below the national average (e.g., Jharkhand 0.30%; Odisha 0.20% [59]). There has been some policy changes taking place at the national level to increase the efficiency of agricultural extension services in India. For example, the National Mission on Agricultural Extension and Technology launched 2014–15 aims to provide a holistic view of agricultural development by including components for technical support and training alongside extension. While it is too early to comment on the achievements of these policy changes, one could easily note that not much has been planned on prioritizing interventions for the socially-marginalized. We have seen, comparing SAS 2003 and SAS 2013 datasets, that no improvement happened in a decade with respect to share of farmers accessing extension services in India. Furthermore, farmers of socially-marginalized castes largely remained outside the reach. We cannot foresee this situation to change in the coming decades without an increased, effective, and focused public investment.

Changing the definition of project success: Alongside increasing the state expenditure on agricultural ER&E, we urge the government interventions to be more inclusive towards farm households of the socially-marginalized castes. There are a number of rural development schemes that ensure priority coverage of marginalized households, such as creation of rural housing [81], delivery of drinking water [82] etc. Even a Constitution Amendment Bill (72nd Amendment Bill of 1991) was introduced in Indian Parliament to provide to ensure participation of scheduled castes and the scheduled tribes in the local governance [83]. Similar policy changes have been largely absent with respect to inclusion of marginalized castes and communities in agrarian development programmes. One of the important recommendations in this connection is to include caste-related variables explicitly in the list of impact indicators of extension programmes. In other words, the success of the extension programmes shall be measured as the number or share of households from the marginalized castes benefitted. A social audit– measuring, understanding, reporting on how resources meet the defined social objectives [84]–can also be made mandatory for these programmes at the grassroot level.

“Ungrouping” the castes: Another major recommendation would be to identify the degree of marginalization among different castes. One of the major findings of our study is that, with respect to farmer participation in public extension system, there is a hierarchically unequal access across caste groups. The OSMCs have not suffered as much SC and ST farmers from the lack of quality extension access, but they too have faced some level of difficulty to access compared to the non-marginalized. There could also be differential access across individual castes within these broad caste groups. Given that the income effects of extension are different for different caste group, agriculture development policies need to be group-specific and governed by specific socio-economic and educational conditions of each caste. Given that the major portion of government investment on ER&E goes for research, leaving little to spend on extension [85], without a clear prioritization agricultural technologies cannot be disseminated in the remote areas, where marginalized castes form a majority of the farming population.

Increased academic focus on caste-related agrarian issues: Alongside policy changes, we may also need a better academic focus on the role of caste on production relations and rural livelihoods. For example, we do not have sufficient information on inter-caste differences in the quality of production resources managed by the farm households, which might also contribute to the lower marginal returns to extension services. The available evidence from India’s neighbouring country, Nepal, suggests that land productivity need not always be lower for socially-marginalized households [86]. However, the situation may vary from region to region. If there are differential quality of production resources across castes, there should be an increased focus to develop and disseminate agricultural technologies to ameliorate these constraints. Historically, the focus of agricultural extension has been on increasing yield with much less attention paid to ecosystem health and natural resource conservation [87]. A differential quality of production resources would imply that focus on the latter would be more relevant for marginalized farm households.

Other recommendations: To increase the reach of agricultural extension among the marginalized, non-traditional approaches could be experimented. One of the recommendations in this regard is about harnessing homophily. In the literature, homophily is shown to facilitate forming of information links and diffusion of technologies [88]. The SAS 2013 dataset does not provide any indication whether the extension agent working with the sample farmers belong to their caste and community, making an estimation of homophily effects impossible.

The final policy recommendation is regarding an explicit inclusion strategy while developing and implementing non-traditional extension methods. Recently, there have been an academic and policy interest toward mobile phone-based information delivery and private extension services. Studies show that the amount, quality and speed of information delivery has improved significantly through some of these interventions [89,90]. However, there exists a knowledge gap on how inclusive these approaches are, given the monetary constraints and differential ability to process information (lower education levels) on part of the socially-marginalized castes and communities. The role of civil society and farmers' organizations in making these new extension approaches socially inclusive needs to be methodically examined.

## Supporting information

S1 Fig Association between non-access due to non-availability of extension services and access probability. (DOCX)

S1 File Data. (XLSX)

S1 Table Caste-differentiated effects of extension contact on crop income: Full regression models. (DOCX)

S2 Table Caste-differentiated effects of extension contact on crop income: District-level fixed effects models. (DOCX)

S3 Table Heterogeneous effects of extension contact on crop income. (DOCX)

S4 Table Regression analysis to test the suitability of variable ‘lack of extension services in the district as the reason for not accessing extension’ as instrument. (DOCX)
