# Supplementary material for: Does caste determine farmer access to quality information?
Source: PLoS One. 2019 Jan 25;14(1):e0210721. doi: 10.1371/journal.pone.0210721 (PMC6347220; doi:10.1371/journal.pone.0210721)
Supplement: S1 Fig — (DOCX) [file pone.0210721.s001.docx]

**S1 Figure. Association between non-access due to non-availability of extension services and access probability**

Note: The dotted line shows the logarithmic trend.
