# Supplementary material for: Does caste determine farmer access to quality information?
Source: PLoS One. 2019 Jan 25;14(1):e0210721. doi: 10.1371/journal.pone.0210721 (PMC6347220; doi:10.1371/journal.pone.0210721)
Supplement: S1 Table — (DOCX) [file pone.0210721.s003.docx]

**S1 Table. Caste-differentiated effects of extension contact on crop income: Full regression model**

**(Part of this table is presented as Table 3)**

|  | **Extension contact measured as a dummy variable** | | | **Extension measured as the frequency of contact** | |
| --- | --- | --- | --- | --- | --- |
|  | **Model 1** | **Model 2** | **Model 1** | | **Model 2** |
| *Caste categories [dummy variables; reference: non-marginalized castes]* |  |  |  | |  |
| Scheduled castes | -25.195^**^ | -7.836^**^ | -25.462^**^ | | -7.946^**^ |
|  | (2.382) | (1.892) | (2.364) | | (1.887) |
| Scheduled tribes | -16.007^**^ | -5.947^*^ | -16.164^**^ | | -5.740^*^ |
|  | (3.080) | (2.463) | (3.037) | | (2.421) |
| OSMC Muslim | -7.768 | -1.311 | -8.129 | | -1.339 |
|  | (6.710) | (4.814) | (6.651) | | (4.760) |
| OSMC non-Muslim | -10.656^**^ | -3.680^*^ | -10.593^**^ | | -3.642^*^ |
|  | (2.392) | (1.826) | (2.326) | | (1.788) |
| *Extension [dummy or frequency] and caste interaction terms* |  |  |  | |  |
| Extension | 28.228^**^ | 12.511^*^ | 3.648^**^ | | 1.905^**^ |
|  | (5.867) | (5.228) | (0.759) | | (0.616) |
| Scheduled castes x Extension | -15.276^*^ | -8.276 | -1.554 | | -0.840 |
|  | (6.457) | (6.117) | (0.947) | | (0.839) |
| Scheduled tribes x Extension | -11.839 | -5.090 | -1.225 | | -0.716 |
|  | (7.464) | (6.268) | (0.985) | | (0.767) |
| OSMC Muslim x Extension | -25.120^*^ | -15.559 | -2.466 | | -1.721 |
|  | (12.133) | (9.186) | (1.683) | | (1.275) |
| OSMC non-Muslim x Extension | -8.474 | -4.242 | -1.150 | | -0.528 |
|  | (6.597) | (5.861) | (1.010) | | (0.794) |
| *Farm-household characteristics* |  |  |  | |  |
| Homestead farming [dummy] |  | -2.163 |  | | -2.030 |
|  |  | (2.131) |  | | (2.114) |
| Size of land owned [ha] per adult equivalent |  | 42.093^**^ |  | | 42.021^**^ |
|  |  | (4.011) |  | | (4.008) |
| Household size [adult equivalents] |  | 10.771^**^ |  | | 10.713^**^ |
|  |  | (0.830) |  | | (0.828) |
| Household head age [years] |  | -0.007 |  | | -0.008 |
|  |  | (0.050) |  | | (0.050) |
| Female household head [dummy] |  | 2.722 |  | | 2.582 |
|  |  | (1.909) |  | | (1.922) |
| Household head education [years] |  | 1.010^**^ |  | | 0.987^**^ |
|  |  | (0.185) |  | | (0.184) |
| Possess owned dwelling [dummy] |  | -1.171 |  | | -0.977 |
|  |  | (3.665) |  | | (3.696) |
| Type of dwelling [1 = bad / kaccha, 2 = medium / semi-pucca 3 = good / pucca] |  | 1.854^*^ |  | | 1.908^*^ |
|  |  | (0.899) |  | | (0.895) |
| *Off-farm income sources [dummy variables]* |  |  |  | |  |
| Livestock production |  | -21.668^**^ |  | | -21.520^**^ |
|  |  | (2.976) |  | | (2.977) |
| Non-farm employment |  | -21.642^**^ |  | | -21.426^**^ |
|  |  | (2.054) |  | | (2.046) |
| Wage employment |  | -19.008^**^ |  | | -18.829^**^ |
|  |  | (1.397) |  | | (1.387) |
| Pension and remittance |  | -19.849^**^ |  | | -19.587^**^ |
|  |  | (2.855) |  | | (2.875) |
| *Region [dummy; reference: North India]* |  |  |  | |  |
| East India | -21.994^**^ | -4.890^*^ | -22.187^**^ | | -5.069^*^ |
|  | (3.476) | (2.315) | (3.438) | | (2.303) |
| South India | -15.223^*^ | 0.437 | -17.065^*^ | | -1.418 |
|  | (7.146) | (7.044) | (7.383) | | (7.242) |
| Semi-Arid Tropics | -4.075 | -0.695 | -3.508 | | -0.716 |
|  | (3.463) | (2.742) | (3.421) | | (2.725) |
| Rest of India | -6.955 | 0.020 | -6.669 | | -0.119 |
|  | (4.339) | (3.292) | (4.269) | | (3.293) |
| *Caste dominance at the district level [dummy]* |  |  |  | |  |
| Non-marginalized castes form a majority [i.e. farmer is from a district where ≥67% belong to the non-marginalized castes] | 11.637 | 12.022^*^ | 11.237 | | 11.717^*^ |
|  | (6.258) | (4.922) | (6.227) | | (4.904) |
| Socially-marginalized castes form a majority [i.e., farmer is from a district where ≥67% belong to the marginalized castes] | 5.145 | 4.080 | 4.695 | | 3.760 |
|  | (2.667) | (2.120) | (2.620) | | (2.105) |
| *Crop types cultivated by the household (dummies; did not include cereals as >90% sample households cultivate a cereal crops)* |  |  |  | |  |
| Legumes |  | -5.140^**^ |  | | -4.982^**^ |
|  |  | (1.479) |  | | (1.468) |
| Sugar |  | 35.987^**^ |  | | 36.105^**^ |
|  |  | (5.271) |  | | (5.230) |
| Spices |  | 15.675^**^ |  | | 15.817^**^ |
|  |  | (3.955) |  | | (3.956) |
| Fruits and nuts |  | 8.229 |  | | 8.118 |
|  |  | (4.450) |  | | (4.392) |
| Roots and starch-yielding crops |  | 0.606 |  | | 0.519 |
|  |  | (2.123) |  | | (2.122) |
| Vegetables |  | 2.190 |  | | 2.203 |
|  |  | (2.166) |  | | (2.183) |
| Oil seeds |  | 1.410 |  | | 1.478 |
|  |  | (1.749) |  | | (1.756) |
| Beverage crops |  | 12.135^*^ |  | | 12.244^*^ |
|  |  | (5.756) |  | | (5.714) |
| Others |  | 20.660^**^ |  | | 20.603^**^ |
|  |  | (2.922) |  | | (2.908) |
| Model intercept | 47.947^**^ | -17.774^**^ | 48.349^**^ | | -17.709^**^ |
|  | (3.757) | (5.801) | (3.702) | | (5.805) |
| *N* | 31,181 | 31,153 | 31,181 | | 31,153 |

Notes: Coefficients are shown with std. errors clustered at the district level in parentheses. Sampling weights given in the SAS 2013 database are employed in the estimation. The dependent variable is crop income. ^*^, ^**^: Statistically significant at 0.05 and 0.01 levels, respectively.

OSMC stands for ‘other socially marginalized communities’.
