# Supplementary material for: Does caste determine farmer access to quality information?
Source: PLoS One. 2019 Jan 25;14(1):e0210721. doi: 10.1371/journal.pone.0210721 (PMC6347220; doi:10.1371/journal.pone.0210721)
Supplement: S2 Table — (DOCX) [file pone.0210721.s004.docx]

**S2 Table. Caste-differentiated effects of extension contact on crop income: District-level fixed effect models**

|  | **Model 1: Extension contact measured as a dummy variable** | **Model 2: Extension measured as the frequency of contact** |
| --- | --- | --- |
| *Caste categories [dummy variables;*  *reference: non-marginalized castes]* |  |  |
| Scheduled castes | -6.4037** | -6.8562** |
|  | (1.4557) | (1.4474) |
| Scheduled tribes | -9.4118** | -9.6463** |
|  | (1.8045) | (1.7926) |
| OSMC Muslim | -1.7100 | -2.0628 |
|  | (3.4047) | (3.3719) |
| OSMC non-Muslim | -2.6995 | -2.9530** |
|  | (1.4800) | (1.4560) |
| *Extension [dummy or frequency] and caste interaction terms* |  |  |
| Extension | 7.3804* | 0.7728 |
|  | (3.4713) | (0.4756) |
| Scheduled castes x Extension | -4.9688 | -0.0473 |
|  | (4.9456) | (0.6849) |
| Scheduled tribes x Extension | -2.9006 | -0.0589 |
|  | (4.8236) | (0.6063) |
| OSMC Muslim x Extension | -8.4400 | -0.6416 |
|  | (7.2589) | (1.0904) |
| OSMC non-Muslim x Extension | 0.6647 | 0.4615 |
|  | (4.1408) | (0.5416) |
| *Farm-household characteristics* |  |  |
| Homestead farming [dummy] | -4.8183* | -4.6598 |
|  | (2.4563) | (2.4594) |
| Size of land owned [ha per adult equivalent] | 42.4823** | 42.4850** |
|  | (2.5560) | (2.5507) |
| Household size [adult equivalents] | 10.6723** | 10.6556** |
|  | (0.6180) | (0.6175) |
| Household head age [years] | 0.0430 | 0.0440 |
|  | (0.0386) | (0.0386) |
| Female household head [dummy] | 2.7309* | 2.6065 |
|  | (1.3823) | (1.3906) |
| Household head education [years] | 0.8482** | 0.8484** |
|  | (0.1582) | (0.1576) |
| Possess owned dwelling [dummy] | -1.4785 | -1.3133 |
|  | (3.1187) | (3.1285) |
| Type of dwelling [1 = bad / kaccha, 2 = medium / semi-pucca 3 = good / pucca] | 1.9265** | 1.9472** |
|  | (0.6769) | (0.6770) |
| *Off-farm income sources [dummy variables]* |  |  |
| Livestock production | -21.2410** | -21.1090** |
|  | (2.8469) | (2.8355) |
| Non-farm employment | -15.6963** | -15.6669** |
|  | (1.6242) | (1.6248) |
| Wage employment | -13.4660** | -13.3937** |
|  | (1.1079) | (1.1070) |
| Pension and remittance | -11.0417** | -10.9043** |
|  | (1.6383) | (1.6406) |
| *Crop types cultivated by the household (dummies; did not include cereals as >90% sample households cultivate a cereal crops)* |  |  |
| Legumes | 0.0210 | 0.0221 |
|  | (1.1970) | (1.1935) |
| Sugar | 38.4365** | 38.6054** |
|  | (3.3117) | (3.3015) |
| Spices | 14.6448** | 14.7372** |
|  | (3.0101) | (3.0049) |
| Fruits and nuts | 10.9529** | 10.8790** |
|  | (3.1265) | (3.1123) |
| Roots and starch-yielding crops | 1.5374 | 1.5340 |
|  | (1.5047) | (1.5058) |
| Vegetables | 4.4031** | 4.4433** |
|  | (1.8067) | (1.7976) |
| Oil seeds | 7.4458** | 7.4280** |
|  | (1.2279) | (1.2254) |
| Beverage crops | 11.7727** | 11.9190** |
|  | (3.1239) | (3.1094) |
| Others | 12.1698* | 12.1665** |
|  | (1.9099) | (1.9120) |
| N | 31,153 | 31,153 |

Notes: Coefficients are shown with std. errors in parentheses. Sampling weights given in the SAS 2013 database are employed in the estimation. The dependent variable is crop income. ^*^, ^**^: Statistically significant at 0.05 and 0.01 levels, respectively.

OSMC stands for ‘other socially marginalized communities’.
