# Supplementary material for: Does caste determine farmer access to quality information?
Source: PLoS One. 2019 Jan 25;14(1):e0210721. doi: 10.1371/journal.pone.0210721 (PMC6347220; doi:10.1371/journal.pone.0210721)
Supplement: S3 Table — (DOCX) [file pone.0210721.s005.docx]

**S3 Table. Heterogeneous effects of extension contact on crop income**

|  | **Farmer is residing in districts where** | | | |
| --- | --- | --- | --- | --- |
|  | **Non-marginalized communities form a majority [>67%]** | **Marginalized communities form a majority [>67%]** | **Non-BPL households form a majority [>67%]** | **BPL households form a majority [>67%]** |
| *Caste categories [dummy variables; reference: non-marginalized castes]* |  |  |  |  |
| Scheduled castes | -5.1346  (3.7042) | -7.8025**  (2.4452) | -6.0012**  (2.1034) | -12.4108**  (4.6188) |
| Scheduled tribes | -2.8575  (12.5718) | -9.9877**  (2.5661) | -7.8566*  (3.2593) | -9.4849*  (4.7015) |
| OBC Muslim | 26.5226**  (8.1836) | -7.1160  (4.9783) | -0.0346  (5.1305) | -14.7092*  (6.4950) |
| OBC non-Muslim | 4.4455  (4.6249) | -1.3758  (2.3943) | -1.8941  (2.1694) | -11.0475*  (4.6953) |
| *Extension [dummy] and interaction terms* |  |  |  |  |
| Extension | 29.3153**  (8.1635) | 3.4102  (5.7948) | 19.0256**  (5.0745) | 1.7772  (7.7530) |
| Scheduled castes x Extension | -45.1821*  (22.4521) | -1.4780  (6.5579) | -18.5658*  (7.9054) | 8.0726  (9.5959) |
| Scheduled tribes x Extension | -15.4520  (34.4024) | 4.0102  (6.6260) | -17.8654*  (7.9715) | 8.7311  (9.9665) |
| OBC Muslim x Extension | -94.5199**  (16.4424) | -2.1773  (9.2885) | -23.5385*  (10.9836) | 1.5231  (12.2741) |
| OBC non-Muslim x Extension | -28.6369  (16.6931) | 8.3070  (6.2227) | -10.3954  (6.3368) | 8.4573  (8.8906) |
| *Farm-household characteristics* |  |  |  |  |
| Homestead farming [dummy] | 0.5060  (12.4744) | -5.3624*  (2.5148) | -3.4914  (3.5736) | -12.7414**  (3.8667) |
| Size of land owned [ha] per adult equivalent | 43.7483**  (12.5589) | 42.1543**  (2.6795) | 56.8940**  (4.2927) | 31.8757**  (4.0786) |
| Household size [adult equivalents] | 14.8712**  (2.9502) | 11.9793**  (0.7718) | 12.4301**  (0.8777) | 10.3267**  (1.6704) |
| Household head age [years] | 0.1198  (0.1559) | 0.0586  (0.0495) | 0.0618  (0.0648) | -0.0231  (0.0960) |
| Female household head [dummy] | -1.6882  (5.5268) | 0.5430  (1.5439) | 3.4810  (1.9685) | -1.9551  (3.2687) |
| Household head education [years] | 1.1598  (0.6471) | 0.9915**  (0.1898) | 0.7860**  (0.2375) | 0.4026  (0.3353) |
| Possess owned dwelling [dummy] | 8.2494  (12.7644) | -6.9492  (3.8986) | 1.1285  (4.6134) | -0.4623  (6.6405) |
| Type of dwelling [1 = bad / kaccha, 2 = medium / semi-pucca 3 = good / pucca] | 0.5544  (4.0246) | 3.0012**  (0.8608) | 2.9696**  (1.1676) | 2.0169  (1.4447) |
| *Off-farm income sources [dummy variables]* |  |  |  |  |
| Livestock production | -63.7783**  (12.4881) | -19.0566**  (3.1396) | -32.1736**  (4.0844) | 0.4083  (7.7926) |
| Non-farm employment | -32.2957**  (5.8679) | -18.1716**  (1.9271) | -20.4041**  (2.4593) | -12.4205**  (3.3747) |
| Wage employment | -28.2397**  (4.2204) | -15.6211**  (1.3195) | -17.7705**  (1.7393) | -14.0062**  (1.8100) |
| Pension and remittance | -26.2009**  (6.1245) | -9.7180**  (1.8616) | -14.9661**  (2.2019) | -0.5297  (5.0480) |
| *Number of observations* | 3,031 | 20,274 | 14,689 | 5,371 |

All models are with district-level fixed effects. Standard errors are shown in parentheses. . ^*^, ^**^ Statistically significant at 0.05 and 0.01 levels respectively.
