# Supplementary material for: Does caste determine farmer access to quality information?
Source: PLoS One. 2019 Jan 25;14(1):e0210721. doi: 10.1371/journal.pone.0210721 (PMC6347220; doi:10.1371/journal.pone.0210721)
Supplement: S4 Table — (DOCX) [file pone.0210721.s006.docx]

**S4 Table. Regression analysis to test the suitability of variable ‘lack of extension services in the district as the reason for not accessing extension’ as instrument**

|  | **Model 1** | **Model 2** |
| --- | --- | --- |
|  | **Access to extension services [dummy variable]** | **Crop income obtained by farm households that did not access extension services** |
| Overall | -0.297*  (0.152) | -0.047  (2.917) |
| Among marginalized castes | -0.419*  (0.194) | -1.349  (3.195) |
| Among non-marginalized castes | 0.037  (0.217) | 9.535  (7.226) |

Notes: Coefficients are shown with std. errors in parentheses. Sampling weights are used in the estimation. The dependent variable of model 2 is measured as thousand Indian rupees. For the complete models, the readers may contact the corresponding author. ^*^: Statistically significant at 0.05 level.
